# AMDock: a versatile graphical tool for assisting molecular docking with Autodock Vina and Autodock4

**DOI:** 10.1186/s13062-020-00267-2

**Published:** 2020-09-16

**Authors:** Mario S. Valdés-Tresanco, Mario E. Valdés-Tresanco, Pedro A. Valiente, Ernesto Moreno

**Affiliations:** 1 Faculty of Basic Sciences, University of Medellin, Medellin, Colombia; 2 Center of Protein Studies, Faculty of Biology, University of Havana, 25 & J, 10400 La Habana, Cuba; 3 Centre for Molecular Simulations and Department of Biological Sciences, University of Calgary, Calgary, Alberta T2N 1N4 Canada; 4 Present address: Donnelly Centre for Cellular & Biomolecular Research University of Toronto, 160 College St, Toronto, ON M5S 3E1 Canada

**Keywords:** AMDock, AutoDock4, AutoDock Vina, AutoDock4Zn, Docking, Graphical user interface

## Abstract

**Abstract:**

AMDock (Assisted Molecular Docking) is a user-friendly graphical tool to assist in the docking of protein-ligand complexes using Autodock Vina and AutoDock4, including the option of using the Autodock4Zn force field for metalloproteins. AMDock integrates several external programs (Open Babel, PDB2PQR, AutoLigand, ADT scripts) to accurately prepare the input structure files and to optimally define the search space, offering several alternatives and different degrees of user supervision. For visualization of molecular structures, AMDock uses PyMOL, starting it automatically with several predefined visualization schemes to aid in setting up the box defining the search space and to visualize and analyze the docking results. One particularly useful feature implemented in AMDock is the off-target docking procedure that allows to conduct ligand selectivity studies easily. In summary, AMDock’s functional versatility makes it a very useful tool to conduct different docking studies, especially for beginners. The program is available, either for Windows or Linux, at https://github.com/Valdes-Tresanco-MS.

**Reviewers:**

This article was reviewed by Alexander Krah and Thomas Gaillard.

## Background

Molecular docking has become a powerful tool for lead discovery and optimization. A large number of docking programs have been developed during the last three decades, based on different search algorithms and scoring functions. Aiming to make these docking programs more user-friendly, especially to beginners, different graphical user interfaces (GUIs) have been developed to assist in the preparation of molecular systems, the execution of the calculations and/or the analysis of the results. Examples of available GUIs (developed mostly for AutoDock [1] and/or Autodock Vina [2]) are AutoDock Tools (ADT), integrated into the PMV graphical package [1], BDT [3], DOVIS [4, 5], VSDocker [6], AUDocker LE [7], WinDock [8], DockoMatic [9], PyMOL AutoDock plugin (PyMOL/AutoDock) [10], PyRx [11], MOLA [12], DockingApp [13] and JADOPPT [14].

We present here a new multi-platform tool, AMDock (Assisted Molecular Docking), whose main advantage over its predecessors is the integration of several valuable external tools within a simple and intuitive graphical interface that guides the users along well-established docking protocols - using either Autodock4 or AutoDock Vina - from system preparation to analysis of results.

### Functionalities and workflow

AMDock integrates functionalities from Autodock Vina and Autodock4, ADT scripts, AutoLigand [15], Open Babel [16], PDB2PQR [17] and PyMOL [18]. For proteins containing a zinc ion in the active site, AMDock has the option of using the specially tailored Autodock4Zn [19] parameters. AMDock is coded in Python 2.7 and is available for Windows and Linux. On Windows, it is packaged together with all the integrated tools, hence no additional software installation is required. On Linux, only Open Babel and PyMOL should be installed (both tools are included in most popular Linux repositories).

**Fig. 1:**
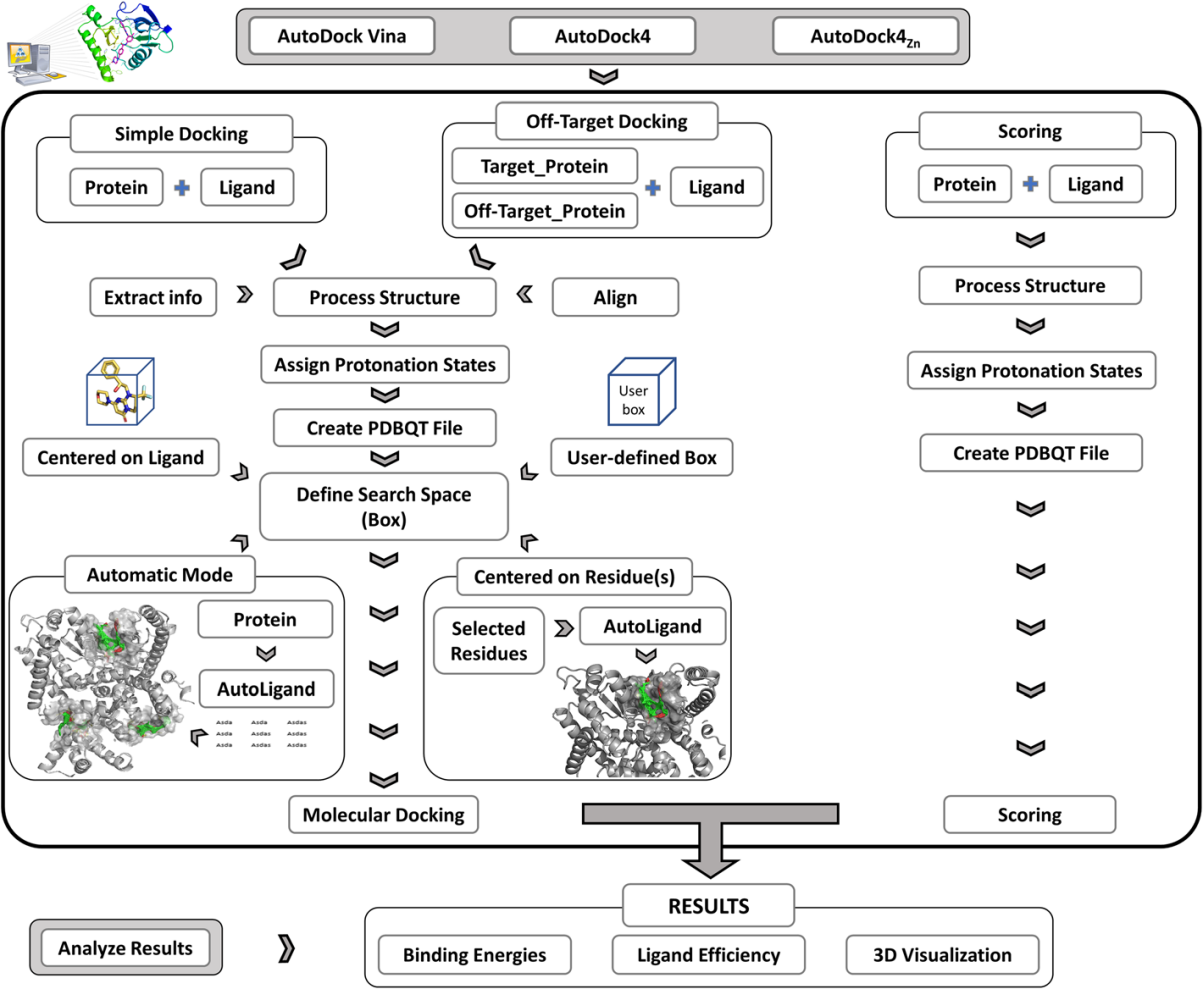
AMDock workflow

The AMDock main window has five tabs: 1) Home, 2) Docking Options, 3) Results Analysis, 4) Configuration, and 5) Info. A summary of AMDock’s functionalities and workflow is presented below (Fig. 1) and discussed afterwards in more detail.

In the “Home” tab, the user can select the docking engine: Autodock Vina or Autodock4, with the additional option of using the Autodock4Zn parameters. Then the user is automatically directed to the “Docking Options” tab, which contains four panels that guide a sequential preparation of a docking simulation.

#### Input files for AMDock

Minimally, the Cartesian coordinates of the ligand and receptor molecules are needed, which can be provided in several common structure formats, e.g. PDB or PDBQT for the protein, and PDB, PDBQT or Mol2 for the ligand. If the protein coordinates come together with a bound ligand, the coordinates of the later are stored and can be used afterward to define the search space.

The program works by following three main steps:

1. *Preparing the docking input files*: First, the user may set a pH value for the protonation of both the ligand (optional, default value 7.4), using Open Babel and the protein (default value: 7.4), using PDB2PQR. Two different docking options are available: a) “simple docking”, for predicting the binding mode of a single protein-ligand complex, and b) “off-target docking”, for predicting the binding poses of a ligand with two different receptors, i.e. the target and the off-target. Finally, the “Scoring” option included in this tab allows to score an already existing protein-ligand complex, using the Autodock Vina, Autodock4 or Autodock4Zn functions. Once the docking or scoring protocol has been selected, the input files are prepared using ADT scripts.
2. *Defining the search space*: Four different approaches can be used to define a box center and dimensions: a) “Automatic” - the program uses AutoLigand to predict possible binding sites and then a box with optimal dimensions is centered on each AutoLigand object,^1^ at each predicted binding site. b) “Center on Residue(s)” - AutoLigand is used to generate an object with a volume in correspondence with the ligand size, using as reference the geometric center of the selected residues. Then, a box with optimal dimensions is centered on the generated object. c) “Center on Hetero” - a box is placed on the geometric center of an existing ligand (if the receptor was given in complex with a ligand), and d) “Box” - the box center and dimensions are defined by the user. The box generated with any of these methods can be visualized in PyMOL and easily modified at the user’s convenience using the new AMDock plugin (adapted from [10]) embedded in the PyMOL menu window.
3. *Running the docking simulations and analyzing the results*: After running the molecular docking calculations (started by clicking the “Run” button), the user will be taken automatically to the “Results Analysis” tab, where the Affinity, Estimated Ki values and Ligand Efficiencies are listed for the different binding poses.

The estimated Ki is a very useful value as it is more related to usually measured experimental parameters, as compared to the affinity. Ligand efficiency (LE), on the other hand, is an important informative parameter when selecting a lead compound [20]. Here, LE is calculated by using the following equation:

1 $$ LE=\frac{-\varDelta G}{HA}, $$

where ΔG is the free energy of binding or the calculated score value and HA is the number of heavy (non-hydrogen) atoms of the ligand. Compounds with LE > 0.3 are highlighted as potential lead compounds [21].

The “Show in PyMOL” button starts PyMOL with a customized visualization of the complex between the receptor and the selected pose (the lowest-energy ligand pose is chosen by default). The resulting data throughout the process is stored in a file (*.amdock), which can be used to examine the results at any time later.

Different docking parameters can be set in the “Configuration” tab, while the “Info” tab, gives access to handy documentation, including a user manual and references.

### Visualization

AMDock relies on PyMOL for visualization at two different stages: 1) setting up the grid box location and dimensions (the search space), and 2) analysis of the docking results. PyMOL is a versatile and user-friendly molecular analysis program which, besides, allows to create high-quality images for publication. We have coded in AMDock several predetermined PyMOL representations for the two stages, selecting the visual design and information that we considered optimal in each case. These predefined representations can be modified by the user within PyMOL.

#### Search space

**Fig. 2:**
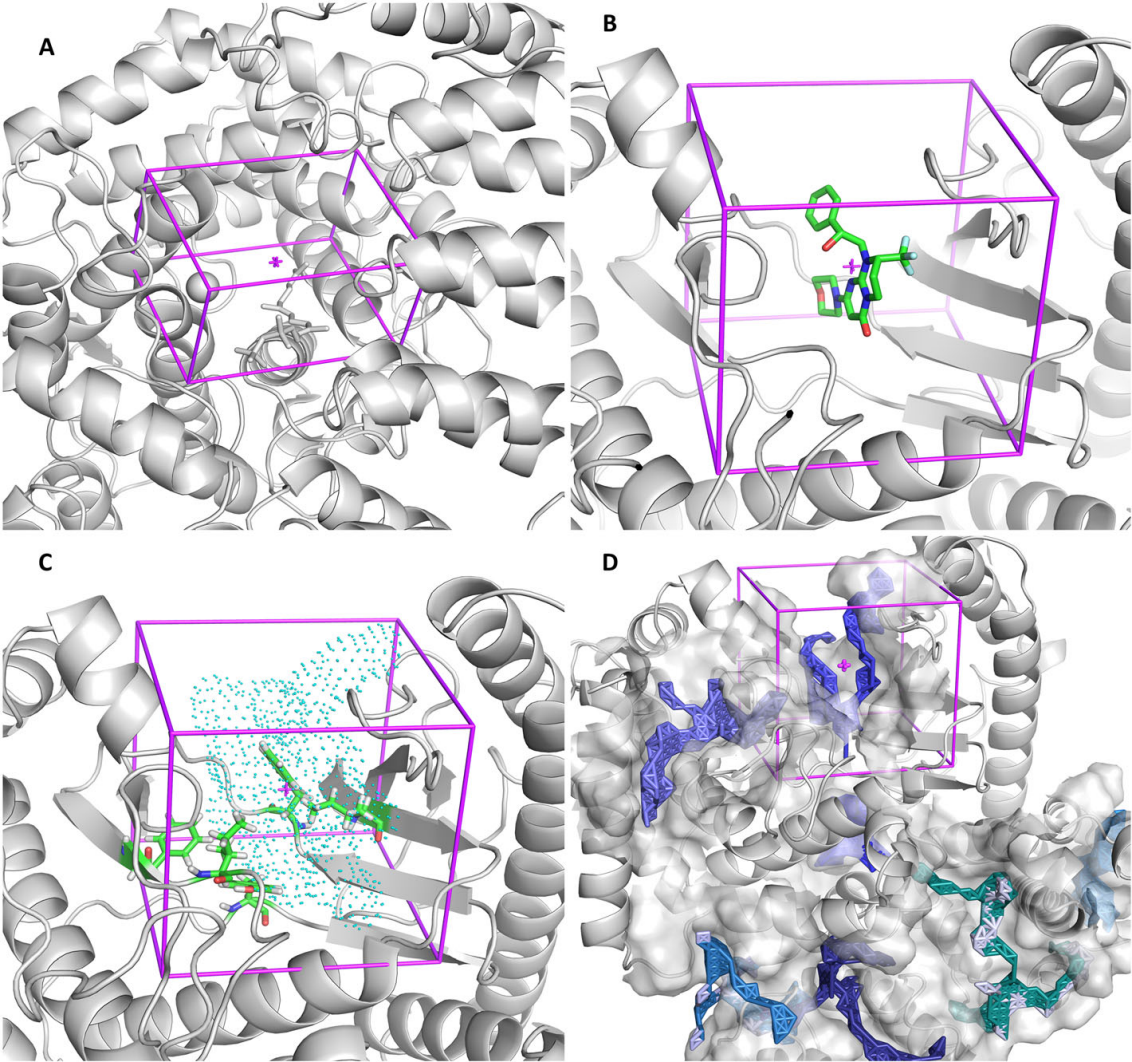
Binding site visualization with PyMOL. **a** User-defined box. This is an example used in tutorials with AutoDock4Zn and farnesyltransferase (hFTase). **b** Centered on Hetero, (**c**) Centered on Residue(s) and (**d**) Automatic mode. Representations B, C and D correspond to Vps34 (PDB: 4uwh)

The predetermined representations (in descending order of complexity, according to the number of elements in the visualization contents) are the following: *1) Box* - a simple representation where the protein under study appears as cartoon, together with the box with the specifications defined by the user (Fig. 2a); *2-Centered on Hetero* - includes the receptor protein (cartoon) and the box with an optimal size centered on the selected previous ligand (sticks) (Fig. 2b); *3-Centered on Residue(s)* - a representation that allows the user to identify the residues that were selected to define the search space. The protein is represented as cartoon, the selected residues as sticks and the AutoLigand object as points. The calculated box is also showed, so that the user can easily check and adjust (if necessary) its position and dimensions (Fig. 2c). *4-Automatic* – Here we intended to create a simplified representation to show all the binding sites predicted by AutoLigand. The protein is in cartoon, each AutoLigand object is represented in sticks, surrounded by a surface constructed on its neighboring residues. Since docking simulations are to be performed for each site predicted by AutoLigand, a box is generated for each site, but showed only for a user-selected site (Fig. 2d). As mentioned above, in any of these variants the box center and size can be easily modified using the AMDock plugin implemented in PyMOL.

#### Results analysis

**Fig. 3:**
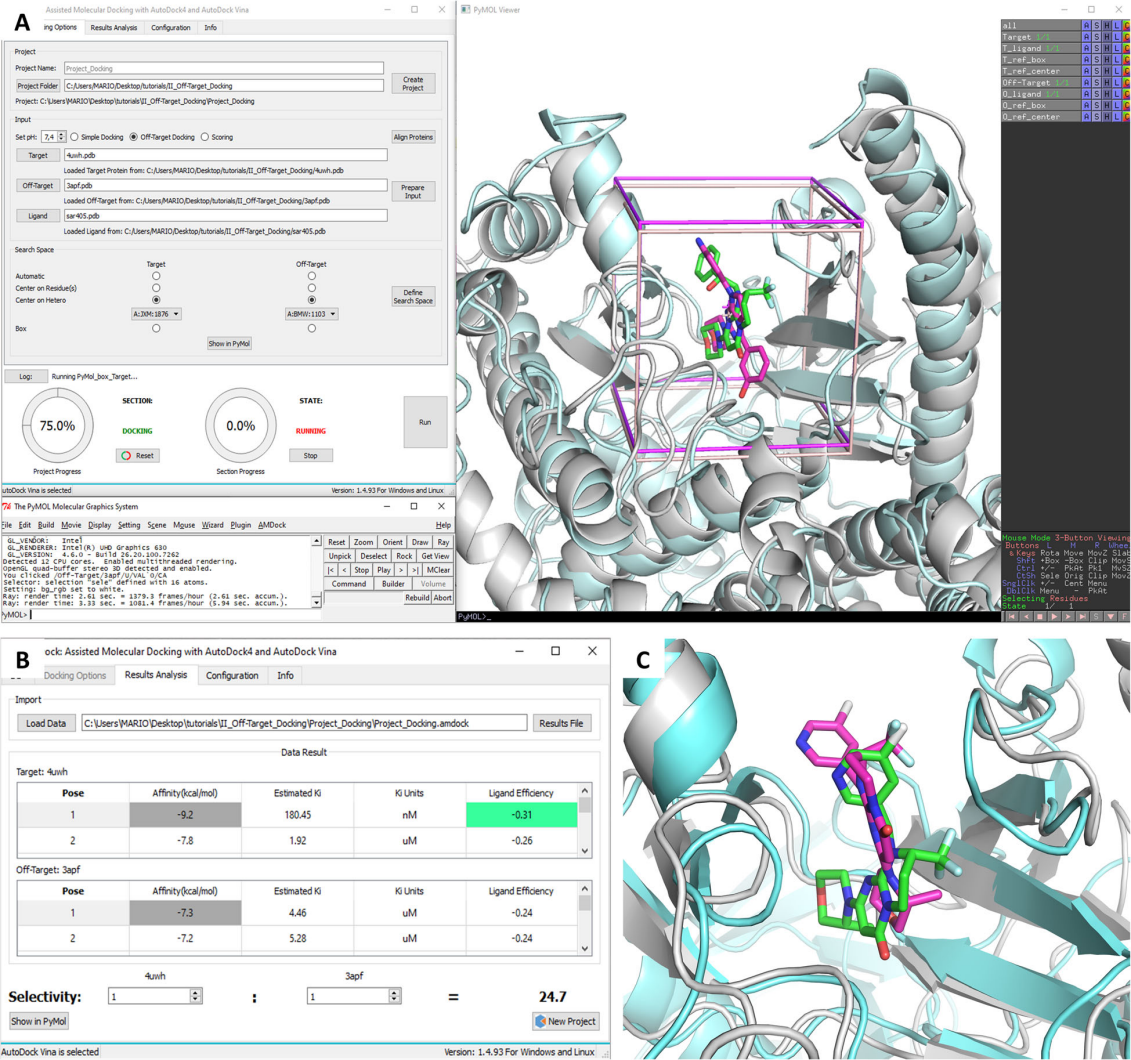
Off-target docking of SAR405. **a** Visualization of the search space for docking, centered on known ligands. **b** Affinity comparison. **c** Superposition of the best pose of SAR405 in complex with PI3Kγ (3apf) (protein in cyan cartoon and ligand in magenta sticks) on the reference complex Vps34-SAR405 (4oys) (protein in gray cartoon and ligand in green sticks)

The protein is represented in cartoon. Each ligand pose is drawn in sticks and its polar contacts with the protein are shown as dashed lines. A similar visualization is also possible for both proteins if the “Off-Target Docking” procedure was chosen (Fig. 3c). This allows a simultaneous comparison of ligand poses for both the target and off-target proteins.

### Case study: SAR405 binding selectivity - PI3Kγ vs. Vps34

Phosphatidylinositol 3-kinase (PI3K) is an enzyme involved in growth, proliferation, motility, survival, and intracellular trafficking [22]. PI3K is also a promising cancer target, with several of its inhibitors being already in the clinical stage. A few of these inhibitors are currently in phase III clinical trials and one of them, alpelisib, recently (May 2019) received the FDA approval for use in the treatment of metastatic breast cancer.

PI3K has several isoforms that are grouped in 3 different classes. Class I includes four different isoforms (α, β, γ and δ), while class III is composed of only one protein, called Vps34 [22]. Because of their sequence and structural similarities, some inhibitors may bind different isoforms, whereas several other inhibitors were designed to be isoform specific. Our research group is currently focused on the identification of PI3K inhibitors with the capability of inhibiting PI3K orthologs found in different pathogenic microorganisms, which express only the ancestral Vps34 isoform. For this purpose, AMDock represents a valuable tool, particularly its “Off-Target Docking” option. Here we demonstrate its use with an exercise that resembles our own research work.

Sar405 is a highly specific inhibitor of Vps34 (IC_50_ = 1.2 nM), while its IC_50_ for other isoforms is > 10^4^ nM [23]. A crystal structure of SAR405 in complex with human Vps34 is available in the Protein Data Bank [24] (PDB code: 4oys). Here we use the human Vps34 as the “Target” receptor, while the PI3K gamma isoform (PDB: 3apf) is used as the “Off-Target” receptor. Both structures contain a bound ligand in the active site, which is convenient for generating the grid box. In the first step, we select the docking program (Autodock Vina) and thereafter a project folder is created in the computer hard disk. After loading both protein structures, we take advantage of their sequence similarity to use the available option of aligning and superimposing their structures using PyMOL, which makes possible defining a common search space and simplifying the subsequent analysis of the docking results. Next, the input files are prepared automatically, which includes protonation of titratable residues, merging of non-polar hydrogens and ion/water removal. The center of the box is defined based on the geometric center of the bound ligands (Fig. 3a), while the size of the box is defined based on the radius of gyration of the ligand to be docked [25], i.e. the SAR405 inhibitor in this case. The initial ligand conformation (its torsion angles) was randomized using ADT.

Once the process is completed, the results show that SAR405 is more selective for Vps34 **(− 9.2** kcal/mol) than for Pi3Kγ (**− 7.3** kcal/mol) as expected (Fig. 3b). The predicted binding pose for SAR405 in Vps34 is close to the crystal geometry (rmsd = 1.9 Å for all ligand atoms, rmsd = 0.5 Å for the ring core). Also, the predicted Ki value for this complex is in the nanomolar range, which agrees with the experimental value. On the other hand, a much higher Ki value is predicted for the Pi3Kγ-SAR405 complex, and the predicted binding pose differs significantly from the crystallographic structure (rmsd = 4.7), as shown in Fig. 3c, which may explain the poor affinity value predicted by AutoDock Vina. This study case has been incorporated as a tutorial in the user manual, which is included in the AMDock installation folder, and the wiki on Github (https://github.com/Valdes-Tresanco-MS/AMDock-win/wiki/4.3-Off-target-docking).

### Discussion

AMDock provides a novel, easy-to-use and versatile interface to work with two molecular docking engines, Autodock4 and Autodock Vina, having different functionalities and characteristics. AMDock should be very useful to researchers with little experience in working with docking programs since no previous knowledge of the particular functioning of these programs is needed. Three different workflows (simple docking, off-target docking and scoring) are included in the AMDock environment. We find the off-target docking procedure particularly helpful for conducting ligand selectivity studies - a critical step in the drug design process.

Preparing the input files in a proper and consistent way, as well as correctly defining the search space, are critical issues when performing molecular docking studies. Several external programs/scripts are integrated into AMDock to allow preparing the input files with minimal effort while keeping control of the process. AMDock uses OpenBabel and PDB2PQR for ligand and receptor protonation, respectively, while the other GUIs mentioned in the Introduction use ADT for both receptor and ligand protonation (with the exception of DockingApp, which uses also OpenBabel for ligand protonation).

To define the search space, AMDock offers several options to set the position of the grid box in different scenarios, while the input ligand is used by default to determine the box optimal dimensions, which decreases the computational cost while optimizing the docking process [25]. In this regard, only ADT and the PyMOL/AutoDock plugin offer some limited options other than a user-defined search space, but in any case the box size must be defined by the user. In some of these GUIs, as in DockingApp, the search space covers the entire receptor, which leads to additional computational costs and possibly compromises the accuracy of the simulations. With other GUIs, the user must use an external applications such as ADT to define the box parameters.

**Fig. 4:**
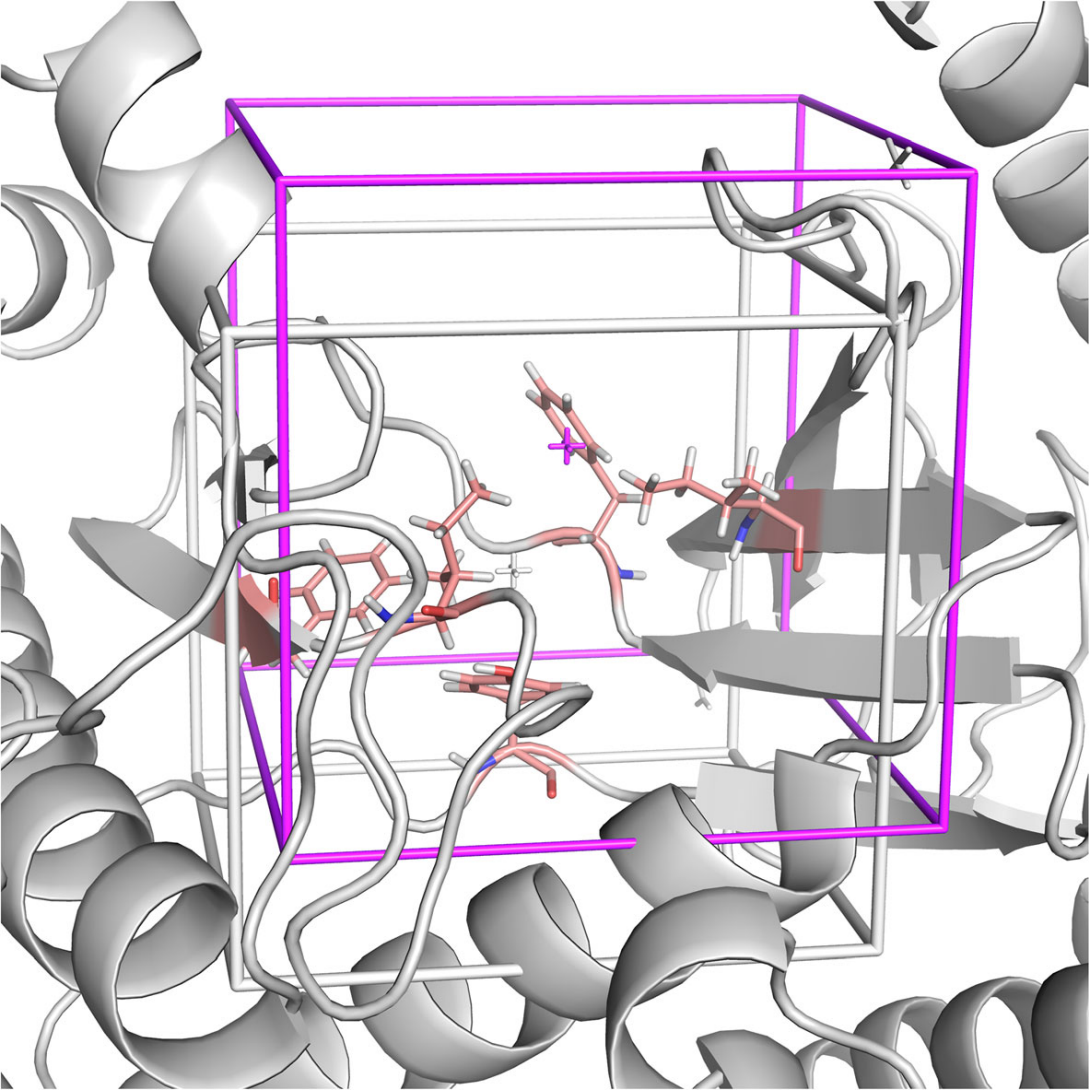
Comparison between a box (white) located at the geometric center of the selected residues (A:ILE:634, A:TYR:670, A:PHE:684, A:PHE:758, A:ILE:760; in salmon) and an a box (magenta) centered on an object generated by AutoLigand from the geometric center of the selected residues. In the later case, the box defines a more optimal ligand sampling space

The “Centered on Residue(s)” option is preferable when the binding site residues are known. With this option, an object placed at the geometric center of the selected residues is generated with AutoLigand on the protein surface. This procedure optimizes both the location and size of the search space. If the box was centered instead on the geometric center of the selected residues, a significant part of it will likely be embedded in the protein, demanding a larger size to cover the needed sampling space (Fig. 4). The “Centered on Hetero” alternative is useful for redocking studies on complexes with crystallographic structures or when studying ligands with similar binding modes (Fig. 2b). The “Automatic” option, on the other hand, is desirable when no information regarding the binding site is available. In this case, an independent docking run is performed for every binding site predicted by AutoLigand (Fig. 2d). This way, the information from the AutoLigand ranking method is combined with that of the docking engine, without making an arbitrary selection of one of the predicted sites. This process is done automatically and the results for each of the predicted binding sites can be visualized in PyMOL. Overall, the definition and visualization of the box involves a minimal effort and can always be modified, thus representing an advantage not only for the novice user but also for experts.

It is worth noting that we standardized the box size to be in Angstroms to avoid commonly occurring errors, as reported in different forums and mailing-lists. These errors arise from the different ways in which the box dimensions are defined in AutoDock (number of points + grid spacing) and Autodock Vina (in angstroms), and may cause the search space to be very small or too large, leading ultimately to inconsistencies in the obtained docking results.

The integration of AMDock with PyMOL represents a significant advantage. Indeed, PyMOL is a widely used molecular viewer with great community support and active development. Within PyMOL, docking results can be analyzed with multiple tools, in particular with the powerful Protein-ligand Interaction Profiler [26]. Other applications such as ADT, PyRx or DockingApp have their own graphical viewers. PyRx and DockingApp offer simple solutions with limited analytical capabilities, while ADT allows only for simple analysis of protein-ligand interactions.

Furthermore, with AMDock it is possible to launch docking simulations for metalloproteins using the AutoDock’s Zn force field, wich is available in ADT only via command line. Its off-target docking option, very useful for drug repurposing studies, is available only in Dockomatic and PyRx (in the later, only in the payment version).

Most of the docking GUIs are focused on virtual screening. Currently, AMDock does not have support for virtual screening, however, we are currently working on its implementation, to make it available in the next program version.

Finally, and since ADT is probably the most widely used docking GUI, we provide a more detailed comparison between AMDock and ADT (Table 1).

**Table 1:** Comparison of AMDock and AutoDock Tools features

Features		AMDock	AutoDock Tools
File formats	**Receptor:**	pdb, pdbqt	pdb, mol2, pdbq, pdbqs, pdbqt, pqr, cif
	**Ligand:**	pdb, pdbqt, mol2	pdb, pdbq, mol2
Protonation	**Receptor:**		
	PDB2PQR	Uses the last version ^1^	Uses PDB2PQR v1.2.1
	pH value adjustment	Yes	No (default 7.0)
	Experimental protonation state	Only if the user enters a protonated structure ^2^	Only histidines or when the user enters a protonated structure
	**Ligand:**		
	Open Babel	Uses the last version^1^	Basic implementation of Open Babel v1.6
	pH value adjustment	Yes	No (default value: 7.0)
Structure manipulation	Flexible Side Chains	Not implemented ^2^	Yes
	Flexible Ligand	Active torsions not implemented ^2^	Yes
	Center Automatic ^3^	Possible binding sites are determined with AutoLigand. Docking is performed for each site.	The user must select a predicted site and prepare the search space. This should be repeated for each site to be tested.
	Center on Residues	Centers the box on an AutoLigand object, calculated for a group of selected residues	Only on a selected atom ^4^
	Center on Hetero	Centers the search space in the geometric center of a heteroatom set found in the defined receptor pdb.	On selected heteroatoms or on a ligand ^5^
	Custom Box	Box coordinates defined by the user.	Box coordinates defined by the user.
	Box Size	Determined from the radius of gyration of ligand, or set by the user ^6^	Defined by the user.
Docking programs	AutoDock4	Yes	Yes
	AutoDock Vina	Yes	Yes
Docking type	Simple	Yes	Yes
	Virtual Screening	No ^2^	No
	Off-target Docking	Yes	No
	Covalent Docking	No ^2^	Yes
	Using Autodock4ZN	Yes	Command line
	Hydrated docking	No ^2^	Command line
Analysis of Results	Simple docking	Yes	Yes
	Virtual Screening	No^2^	Yes
	Off-target docking	Yes	No
	Covalent docking	No ^2^	Yes
	Autodock4 ZN docking	Yes	Yes
	Hydrated docking	No ^2^	Yes
Graphical Visualization	Engine	PyMOL	Python Molecular Viewer
	Capacity	All the options included in PyMOL	Protein-ligand interactions and cluster manager for AutoDock4 results
	Publication-quality images	Easy high-resolution and custom image generation	Easy low-resolution image generation. Difficult high-resolution image generation
	Maintenance	Active development	Inactive
Programming	Python base	Python 2.7.15^7^	Python 2.6 (Inactive development)
Easy to use ^8^	Docking preparation	1	4
	Analysis of Results	2	3
	GUI simplicity	1	4
	Process Log	1	4
	Installation	2	2
	Platform	Linux and Windows	Linux, Windows and Mac

^1^ Last version with Python 2.x support

^2^ Will be available in the next release

^3^ A common alternative is to do the so-called blind docking, in which the search space is defined to cover the entire receptor. This involves an increasing in the sampling number so as not to compromise the accuracy of the docking, which leads to an increase in computational cost. Additionally, it can introduce false positives by sampling sites with a different nature than the binding site. Results are usually questionable due to the non-convergence of the scoring functions

^4^ We describe the advantages of the method used in AMDock concerning this selection (Fig. 4)

^5^ It is possible to select an atom only if the heteroatoms of the receptor have not been removed. After that, these atoms must be removed and the receptor should be redefined. Another possibility is entering a set of heteroatoms and directly select the option “center on ligand”. Both options have limitations and need a deeper understanding of the ADT program

^6^ We describe the advantages of the method used in AMDock

^7^ Next version in python 3.x under development (https://github.com/Valdes-Tresanco-MS/AMDock-win-py3)

^8^ Our own evaluation using a 1–5 scale, where 1 is very easy and 5 is very difficult

### Conclusions

AMDock is a user-friendly GUI that works in a highly intuitive and interactive manner, allowing to perform molecular docking studies with Autodock4 and AutoDock Vina with a minimal setup effort. These characteristics make AMDock an attractive tool also for teaching purposes. AMDock gathers features and procedures that are not present in other similar programs. It includes recent developments in AutoDock, such as the Autodock4Zn parameterization. For our group, AMDock has been very useful for estimating the selectivity profile of different PI3K inhibitors over orthologous proteins in several microorganisms. Further developments (hydrated ligand, covalent docking and virtual screening) will be included as docking options in future versions.

## Reviewers’ comments

### Reviewer 1, Alexander Krah

Summary: Valdés-Tresanco et al. describe in their manuscript entitled “AMDock: A versatile graphical tool for assisting molecular docking with Autodock Vina and Autodock4” the implementation of several molecular modelling tools in a graphical user interface, which allows to setup and perform docking simulations with Autodock4 or Autodock Vina. I think the tool is interesting and may allow individuals who just begin with molecular docking to quickly go through the whole process. However, I would like to ask for clarifications.

Response: We thank Dr. Krah for his positive comment. Below we respond point-by-point his questions and critical comments.

*Mayor recommendations*

*1) Could the authors describe in more detail, how the pKa for titratable groups of the ligand is calculated (e.g. carboxylate or amine groups)?*

Response: Ligand hydrogen atoms are added either with Open Babel (default) or ADT. Open Babel uses a set of fragments with known pKa to estimate the protonation state of the ligand (Open Babel Documentation). ADT adds hydrogens depending on the atom type, its valence and minor chemical group considerations based on a reimplementation of the PyBabel v1.6 module of Open Babel (PyBabel documentation in ADT). The user can decide whether to use AMDock's internal tools or entering a protonated ligand. In the later case hydrogens will not be added.

This point was also a concern of Reviewer 2. We have added a statement on the optional protonation choice in the subsection “Functionalities and workflow”. It reads: “… (optional, default value 7.4), using Open Babel …”

*2) Can the user set experimentally know protonation states for protein residues? 3) Can protein residues be set flexible, as this possibility is incorporated in ADT/Autodock Vina.*

Response: At the moment these functionalities are not available. However, they will be incorporated in the next AMDock version (development version in https://github.com/Valdes-Tresanco-MS/AMDock-win-py3)

*4) The authors report as an example of an off-target binding prediction, resulting in a higher score for the target than the off-target. Could the authors test two more examples, if targets with required structural and biophysical information can be found:*

*a) different inhibitors binding in the same range to the same protein bound to the same site?*

Response: Three docking exercises included either in the documentation or the manuscript involve three inhibitors having similar reported IC_50_ values and the same protein crystallographic structure (PDB code 4UWH, a Vps34-inhibitor complex)

- The re-docking exercise (https://github.com/Valdes-Tresanco-MS/AMDock-win/wiki/4.2.2.1-re-Docking-experiment) uses the crystallographic ligand from 4UWH: compound No. 4 (IC_50_ = 3nM) described by Pasquier *et al*., 2014.

- In the “similar docking ligand” exercise (https://github.com/Valdes-Tresanco-MS/AMDock-win/wiki/4.2.2.2-Docking-a-similar-ligand), we use inhibitor No. 31 (IC_50_ = 2 nM, https://www.rcsb.org/ligand/7A5) also described by Pasquier *et al*., 2014.

- Finally, inhibitor SAR405 (IC_50_ = 1.2nM, https://www.rcsb.org/ligand/1TT) is described as a case study in the manuscript (https://github.com/Valdes-Tresanco-MS/AMDock-win/wiki/4.3-Off-target-docking).

All inhibitors have similar IC_50_ values and bind to Vps34 in the same binding site. In all cases, AutoDock Vina was able to reproduce the crystallographic complex, with affinities of -9.2, -8.7 and -9.2 kcal/mol, respectively, and estimated Ki values in the nanomolar range.

It is important to mention that our case studies are only representative examples of the methodologies implemented in AMDock. As known from the literature, the accuracy of the prediction depends on several structural factors, *i.e.*, the protonation state, the quality of the receptor structure, the particular side chain orientations in the binding site (related to the induced fit effect), among others. AMDock provides a platform for preparing docking files and optimize the search space. However, it does not influence the predictability of the program used to perform the docking. As we remark in the documentation, the interpretation of the results must be based on empirical/experimental evidence, which must be carefully studied by the user.

*b) different inhibitors binding in the same range to the same target bound to a different (potentially allosteric) site?*

Response: A new tutorial has been included in the AMDock wiki (https://github.com/Valdes-Tresanco-MS/AMDock-win/wiki/4.5.2-Docking-to-allosteric-binding-sites) for such a system.

*5) How does the program perform in comparison with Autodock Vina and other tools, which incorporate Autodock Vina?*

Response: To date, most tools that incorporate Autodock Vina are discontinued. We intend to provide a tool where routinary docking experiments (simple docking, off-target docking, redocking, etc.) can be performed with minimal effort. In terms of speed, AMDock does not provide a better performance since it uses the standard Autodock Vina engine. However, the integration of several externals tools to prepare the docking files and define/optimize the search space saves time while avoiding commonly-made errors.

### Reviewer 2, Thomas Gaillard

Summary: The manuscript of Valdés-Tresanco, Valdés-Tresanco, Valiente, and Moreno presents AMDock, a graphical tool aimed at facilitating molecular docking with Autodock Vina and Autodock4. AMDock integrates external programs (Open Babel, PDB2PQR, AutoLigand, ADT scripts). Molecular visualization is performed with PyMOL. The program is available for Windows and Linux and is distributed on Github (https://github.com/Valdes-Tresanco-MS). Overall, the work presented is valid and well written. The significance and originality are however limited. There are indeed already many existing GUIs facilitating docking calculations. In particular, the AutoDock Tools (ADT) interface to Autodock Vina and Autodock4 is well conceived and offers more functionalities than AMDock. The authors do not provide a detailed comparison of AMDock and other GUIs. To my opinion, the manuscript is not convincing at demonstrating how AMDock is advantageous, at least on some points, compared to existing tools. At last, some methodological points need to be clarified in the case study.

Response: We appreciate Dr. Gaillard’s comments and agree with him in that other GUIs (ADT in particular) offer several functionalites that are not available in AMDock. Our goal, however, was not to outperform ADT, but to provide a simple, easy-to-use tool with a maximal optimization of the docking procedures. We are currently working on expanding AMDock’s capabilities, though we do believe that in its current state the program can be useful in many aspects.

*Mayor recommendations*

*1) A list of other docking GUIs is provided by the authors in the Background section. A useful addition to the manuscript, maybe in the Discussion section, would be a comparative table of functionalities for these GUIs and AMDock.*

Response: In the Discussion section we now address in more details the comparison between AMDock and other tools mentioned in the Introduction section. Please see the response to next point (2).

*2) In particular, the authors should discuss what are the advantages of their tool with respect to AutoDock Tools (ADT), which is probably the most widely used GUI for Autodock and Vina. ADT also offers many options for ligand and receptor preparation, docking input file preparation, launching docking, results analysis, etc. In addition, ADT includes its own visualization interface, whereas AMDock depends on an external program (PyMOL). - The authors claim that the main advantage of their tool is "the integration of several valuable external tools within a simple and intuitive graphical interface that guides the users along well-established docking protocols - using either Autodock4 or AutoDock Vina - from system preparation to analysis of results" (p3). This is rather vague and it would be helpful to clarify which of these external tools offer a unique advantage over existing GUIs.*

Response: We have included in the Discussion section a more comprehensive comparison between AMDock and ADT – the new Table 1. We would like to emphasize that our aim in developing AMDock is not to replace ADT, but to create a helpful complement. From our perspective, ADT can be a bit difficult to manipulate for non-expert users. On the other hand, we continue working to make AMDock a more versatile and robust tool. Finally, it is worth mentioning that, to our knowledge, ADT development is not currently active.

*3) In particular, a list of external tools integrated by AMDock is provided (p3). Most of these tools are described in the manuscript, at the exception of OpenBabel, whose role is not discussed.*

Response: Please see the response to point 1 by Reviewer 1.

*4) I may have missed the point but it is not clear to me what this procedure brings more than two standard dockings with the target and off-target receptors.*

Response: In principle, they are two separate standard dockings. However, we do believe that carrying out docking on both receptors at the same time is an advantage. As pointed out by the Reviewer, the comparison conditions should be similar in both cases. Here, we allow the user to protonate both receptors to the same pH, determine the optimal search space for both receptors, run the docking simulations with the same selected program and analyze the results in a simple comparative format. Furthermore, the user can superimpose the receptors and obtain a visual comparison of both the search space and the docking results. Carrying out all these steps separately requires additional effort and may lead to errors. This type of docking exercise is the basis for inverse virtual screening used primarily in drug repurposing. We do intend to implement this feature in future AMDock versions.

*5) In the case study, the authors are docking an inhibitor on target and off-target receptors and find that the inhibitor indeed prefers the target. In such tests, it is important to ascertain the fairness of the comparison. It is not clear how the initial conformation of the inhibitor is chosen. If the docking is in any way biased in favor of the known pose, the comparison with the off-target is not fair. The authors need to make sure that the initial conformation is randomized and that a sufficiently large search box is used.*

Response: We now describe in more details the starting conditions, stating that the initial ligand conformation was randomized. The search space has the same dimensions for both receptors, since the box size depends on the radius of gyration of the ligand (SAR405), which is a constant parameter in this exercise. The center of the box is determined by the geometric center of the co-crystallized ligands in both complexes. As can be observed in Figure 3A, the two boxes enclose their corresponding binding sites with sufficient margins, so no bias is introduced for any of the receptors.

*Minor recommendations*

*1) PyMOL is importantly used by AMDock. Could the authors give information on the compatibility of AMDock with the different versions of PyMOL?*

Response: Previously, AMDock worked with PyMOL 1.8.5. Recently, we verified that the AMDock plugin is compatible also with PyMOL v2.x. The current Windows distribution works with PyMOL version 2.1. In Linux, AMDock works with any installed PyMOL version.

PyMOL is a robust and widely used visualization program. Currently, its use as an external program in AMDock implies limitations on its full exploitation. We are currently working on its incorporation as a native viewer for AMDock. This will be a significant advantage since it will allow a direct exchange of information between AMDock and PyMOL (for example, to manipulate the receptor and the ligand, for active visualization of molecule preparation, creation, visualization and modification of the search space, flexible side chain selection, etc.).

2) Why version 2.7 of Python is used and not 3.*? Note that 2.7 will not be maintained past 2020

Response: AMDock is programmed in Python 2.7 to guarantee full compatibility with third-party programs (e.g.: AutoDockTools, PDB2PQR, AutoLigand, etc.). Some of these programs have been recently updated to Python 3. ADTs are critical to prepare the input files for docking with AutoDock or AutoDock Vina. We have recently created a version of ADT in Python3 available here (https://github.com/Valdes-Tresanco-MS/AutoDockTools_py3). We plan to migrate all the code to Python 3 in future versions (a developmental version in Python3 is available here (https://github.com/Valdes-Tresanco-MS/AMDock-win-py3).

*3) In the case study, a step consists in "aligning and superimposing their structures". It is not clear to me if it is an AMDock or a PyMOL functionality*

Response: It is done with PyMOL. A remark was added in the Case study section.

*4) The concept of an "AutoLigand object" is used in the manuscript but not defined*

Response: It is now defined in a footnote.

*5) What "previous ligand" means on p7 l1?*

Response: We define "previous ligand" as any fragment of heteroatoms that appear co-crystallized with the protein in the PDB file. Ideally, the user should leave only the atom sets of interest, *i.e*. inhibitors, fragments or substrates found in the crystallographic structure. If a protein-ligand complex is introduced as the receptor, the coordinates of the ligand(s) are also stored. These coordinates can be used in the search space determination option called “Centered on Hetero”, as explained in the manuscript.

## Data Availability

The manual as well as tutorial files are included in the program installation folder. Additionally, information such as common errors, frequently asked questions, etc., can be found in the AMDock repository and the following mailing-list: (https://groups.google.com/forum/#!forum/amdock).

## Abbreviations

GUI: Graphical user interface
ADT: AutoDockTools
PMV: Python molecular viewer
AMDock: Assisted Molecular Docking
PI3K: Phosphatidylinositol 3-kinase
LE: Ligand efficiency

## Footnotes

1 AutoLigand Object is equivalent to the term “envelope” used by the authors of AutoLigand and consists of a representation of a contiguous region in space, filled with points that represent potential atomic centers for ligand atoms [15]
